# Development of a single-shot CCD-based data acquisition system for time-resolved X-ray photoelectron spectroscopy at an X-ray free-electron laser facility

**DOI:** 10.1107/S1600577513028233

**Published:** 2013-12-10

**Authors:** Masaki Oura, Tatsuya Wagai, Ashish Chainani, Jun Miyawaki, Hiromi Sato, Masaharu Matsunami, Ritsuko Eguchi, Takayuki Kiss, Takashi Yamaguchi, Yasuhiro Nakatani, Tadashi Togashi, Tetsuo Katayama, Kanade Ogawa, Makina Yabashi, Yoshihito Tanaka, Yoshiki Kohmura, Kenji Tamasaku, Shik Shin, Tetsuya Ishikawa

**Affiliations:** a Research Infrastructure Group, RIKEN SPring-8 Center, 1-1-1 Kouto, Sayo-cho, Sayo-gun, Hyogo 679-5148, Japan; b Excitation Order Research Team, RIKEN SPring-8 Center, 1-1-1 Kouto, Sayo-cho, Sayo-gun, Hyogo 679-5148, Japan; c Harima Branch, Institute for Solid State Physics, The University of Tokyo, 1-1-1 Kouto, Sayo-cho, Sayo-gun, Hyogo 679-5198, Japan; d Institute for Solid State Physics, The University of Tokyo, Kashiwanoha 5-1-5, Kashiwa, Chiba 277-8581, Japan; e UVSOR, Institute for Molecular Science, Myodaiji, Okazaki, Aichi 444-8585, Japan; f Research Laboratory for Surface Science, Okayama University, Okayama 700-8530, Japan; g Graduate School of Engineering Science, Osaka University, Toyonaka, Osaka 560-8531, Japan; h XFEL, JASRI, 1-1-1 Kouto, Sayo-cho, Sayo-gun, Hyogo 679-5198, Japan; i XFEL Research and Development Division, RIKEN SPring-8 Center, 1-1-1 Kouto, Sayo-cho, Sayo-gun, Hyogo 679-5148, Japan; j RIKEN SPring-8 Center, 1-1-1 Kouto, Sayo-cho, Sayo-gun, Hyogo 679-5148, Japan

**Keywords:** data acquisition system, shot-to-shot measurement, soft X-ray photoelectron spectroscopy, hard X-ray photoelectron spectroscopy, X-ray free-electron laser

## Abstract

In order to utilize high-brilliance photon sources, such as X-ray free-electron lasers (XFELs), for advanced time-resolved photoelectron spectroscopy (TR-PES), a single-shot CCD-based data acquisition system combined with a high-resolution hemispherical electron energy analyzer has been developed. The system’s design enables it to be controlled by an external trigger signal for single-shot pump–probe-type TR-PES. The basic performance of the system is demonstrated with an offline test, followed by online core-level photoelectron and Auger electron spectroscopy in ‘single-shot image’, ‘shot-to-shot image (image-to-image storage or block storage)’ and ‘shot-to-shot sweep’ modes at soft X-ray undulator beamline BL17SU of SPring-8. In the offline test the typical repetition rate for image-to-image storage mode has been confirmed to be about 15 Hz using a conventional pulse-generator. The function for correcting the shot-to-shot intensity fluctuations of the exciting photon beam, an important requirement for the TR-PES experiments at FEL sources, has been successfully tested at BL17SU by measuring Au 4*f* photoelectrons with intentionally controlled photon flux. The system has also been applied to hard X-ray PES (HAXPES) in ‘ordinary sweep’ mode as well as shot-to-shot image mode at the 27 m-long undulator beamline BL19LXU of SPring-8 and also at the SACLA XFEL facility. The XFEL-induced Ti 1*s* core-level spectrum of La-doped SrTiO_3_ is reported as a function of incident power density. The Ti 1*s* core-level spectrum obtained at low power density is consistent with the spectrum obtained using the synchrotron source. At high power densities the Ti 1*s* core-level spectra show space-charge effects which are analysed using a known mean-field model for ultrafast electron packet propagation. The results successfully confirm the capability of the present data acquisition system for carrying out the core-level HAXPES studies of condensed matter induced by the XFEL.

## Abstract 2

A single-shot CCD-based data acquisition system for time-resolved photoelectron spectroscopy using an X-ray free-electron laser has been developed. The basic performance of the system is demonstrated using XFEL-induced and synchrotron-radiation-induced Ti 1*s* core-level spectroscopy.

## 1. Introduction   

With the advent of recent high-brilliance photon sources such as third-generation synchrotron radiation, high-harmonic generation (HHG) and free-electron laser (FEL) sources, time-resolved photoelectron spectroscopy (TR-PES) has become one of the fascinating spectroscopic techniques providing ‘real time’ direct information on the ultrafast (picosecond and femtosecond) dynamics in electronic structure of condensed matter. Most of the TR-PES experiments performed up to the present rely upon a pump–probe measurement scheme which combines femtosecond optical and HHG (or FEL) laser pulses, where the temporal evolution of selected electronic states can be determined as a function of the delay between optical pump and HHG (or FEL) probe pulses.

Early studies on TR-PES were mainly based on the utilization of a β-BaB_2_O_2_ crystal to produce a frequency-doubled UV pulse as HHG probe from a Ti:sapphire laser (Aeschlimann *et al.*, 2000 ▶; Bauer & Aeschlimann, 2002 ▶; Perfetti *et al.*, 2006 ▶, 2007 ▶, 2008 ▶; Schmitt *et al.*, 2008 ▶; Azuma *et al.*, 2009 ▶). The recent trend for sources in TR-PES has been to adopt the harmonic generation process using a rare gas (Lewenstein *et al.*, 1994 ▶; Haight & Peale, 1994 ▶), which can give significantly high intensities even for much higher harmonics (Dakovski *et al.*, 2010 ▶; Ishizaka *et al.*, 2011 ▶; Dachraoui *et al.*, 2011 ▶; Rohwer *et al.*, 2011 ▶; Petersen *et al.*, 2011 ▶). Hence, it provides us with an opportunity to study the photo-induced transient electronic structure of condensed matter even by means of core-level TR-PES, thereby allowing element-specific studies (Ishizaka *et al.*, 2011 ▶; Dachraoui *et al.*, 2011 ▶; Siffalovic *et al.*, 2002 ▶). Nowadays, the HHG source can cover the energy range of the water window (Chen *et al.*, 2010 ▶), but the resultant light is not suitable for spectroscopy due to limitations of tunability, number of photons per pulse and its broad bandwidth, although its time structure can be as narrow as 11 attoseconds full width at half-maximum (FWHM).

The emergence of FEL sources may lead to a breakthrough in these limitations for applying such femtosecond tunable light pulses to core-level TR-PES in materials science. Pietzsch *et al.* (2008 ▶) and Hellmann *et al.* (2010 ▶, 2012 ▶) have carried out pioneering work on TR-PES with monochromated FEL probe pulses at FLASH (Ackermann *et al.*, 2007 ▶), but to date there has been no other report on photoemission spectroscopy for condensed matter using a FEL source. This is perhaps due to some difficulties concerning pulse-to-pulse intensity fluctuations and wavelength change inherent to the self-amplified spontaneous emission (SASE) process. Furthermore, FEL-induced vacuum space-charge effects (Hellmann *et al.*, 2012 ▶) cause an additional difficulty in performing TR-PES with a FEL probe pulse. Although the difficulty concerning wavelength change is reduced by adopting the monochromator, it is better that a data acquisition (DAQ) system with shot-to-shot measurement scheme is synchronized to the recording system for the corresponding FEL pulse properties. In previous studies (Pietzsch *et al.*, 2008 ▶; Hellmann *et al.*, 2010 ▶, 2012 ▶) photoemission spectra were acquired using a shot-to-shot measurement system with a gated CCD camera synchronized to the FEL macropulse with repetition rate of 5 Hz. In order to realise the shot-to-shot measurement system synchronized with a FEL single pulse of 10 Hz repetition rate without counting-loss we have developed a single-shot DAQ system for TR-PES by using a CCD-based high-resolution hemispherical electron energy analyzer to advance spectroscopic studies on transient phenomena in condensed matter. The system is aimed at performing TR-PES with a high-brilliance FEL source operating at a low repetition rate and designed to be controlled by an external trigger signal, *e.g.* a master trigger signal from the accelerator.

The purpose of this paper is to describe the single-shot CCD-based DAQ system and demonstrate its feasibilities for the shot-to-shot measurement scheme with the aid of the characterizing experiments using soft and hard X-ray synchrotron radiation beams. The system has also been applied to hard X-ray photoelectron spectroscopy (HAXPES) at an X-ray free-electron laser (XFEL) facility, SACLA (Ishikawa *et al.*, 2012 ▶).

## 2. Photoelectron spectroscopy apparatus combined with a single-shot DAQ system   

### 2.1. Hardware and software for the single-shot DAQ system   

The single-shot DAQ system, which can be triggered by an external signal and is developed for the CCD-based hemispherical electron energy analyzer, consists of a multifunction DAQ device, *e.g.* National Instruments (NI) PCIe-6363 (National Instruments, 2012 ▶), and a conventional CCD camera (480 × 433 pixel arrays). The maximum readout speed of the present CCD camera is 70 frames s^−1^ with a FireWire (IEEE 1394) connection. The system can be used for acquiring an energy-analyzed photoelectron image in pump–probe-type TR-PES for materials science using the FEL source.

In the present system the DAQ device can work as an analog-to-digital converter (ADC), a digital-to-analog converter (DAC) and/or a counter. The sampling rate of ADC channels is 2 MS s^−1^ for one analog input. Output from the DAC channel is configured to be a transistor-transistor-logic (TTL) pulse for multipurpose use, and the counter channel can be used to monitor the voltage output from any electronics by combining with a voltage-to-frequency converter. The system can be used for single-shot measurement and is designed to proceed iteratively at 10 Hz without counting-loss in the shot-to-shot measurement scheme.

The software to control the NI PCIe-6363 is entirely coded using *LabVIEW* and is also configured in order to facilitate the external control of the electron analyzer, *e.g.* VG SCIENTA R4000-10kV, with the aid of the SESWrapper library (VG Scienta, 2008 ▶). In the software, ‘single-shot image’, ‘shot-to-shot image’ and ‘shot-to-shot sweep’ modes are packaged. Actually, shot-to-shot image mode corresponds to an iteration of the single-shot image mode. The shot-to-shot sweep mode, on the other hand, additionally includes a kinetic energy sweep of the hemispherical analyzer. Two-dimensional image data of the energy-analyzed photoelectrons are sequentially acquired *via* the CCD camera, and the data are immediately transferred to the stacked memory and finally stored in mass storage. As for the data storage, there are two storage modes for shot-to-shot image mode. One is the image-to-image storage mode, and the other is the block storage mode. In the block storage mode the DAQ system accumulates consecutive images in the stacked memory and, when the number of images reaches a certain number, *e.g.* 1000, all the images in the stacked memory are stored in mass storage. During the data storage of the block storage mode, *e.g.* typically a few seconds, a new image accumulation is inhibited until the end of the storage. Besides the image data, various information, such as the intensity and wavelength of each exciting photon pulse, can be acquired simultaneously by using the NI PCIe-6363. Therefore pulse-to-pulse intensity fluctuations and wavelength changes inherent to the SASE process can be corrected. Offline analysis software is also prepared to analyze the enormous amount of image data in order to convert them to kinetic energy spectra.

### 2.2. Achieved performance of the single-shot DAQ system   

We have measured the typical processing time of the single-shot DAQ system. The external trigger signal is generated by using a conventional pulse-generator. When the NI PCIe-6363 is triggered, the hardware starts the A/D conversions and counter, where the accumulation time for the counter has been set to be 20 ms. Simultaneously, a command to control the electronic shuttering of the CCD camera is issued. In the performance test the CCD camera has been set to acquire a still image. After a certain exposure time, typically 14 ms, image data from pixel arrays are read out and immediately transferred to the stacked memory on the PC, and the data are finally stored in mass storage. When the data transfer to the stacked memory is finished, the CCD and DAQ device can be cleared and the DAC channel outputs the TTL pulse instantly for multipurpose use. When the repetition rate of the external trigger is higher than the processing speed of the system, a new trigger signal is inhibited during the processes mentioned above and is treated as counting-loss.

**Figure 1:**
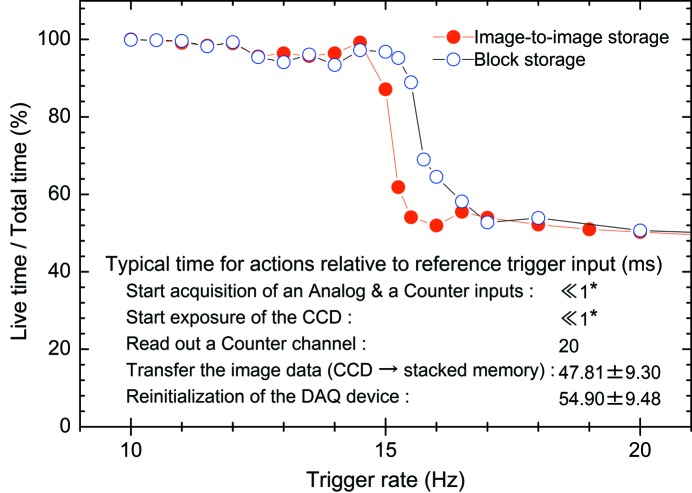
Counting efficiency of the present DAQ system for image-to-image storage mode (red closed circles) and block storage mode (blue open circles) measured as a function of trigger rate. In the lower half of the figure, typical times relative to the reference time of the trigger arrival for actions of shot-to-shot image mode are also shown. *Time scale is in the microsecond range. The minimum time, which can be measured using *LabVIEW*, is 1 ms.

In order to estimate the optimum frequency enabling the data acquisition without counting-loss, we have measured the relation between counting efficiency and trigger rate for the image-to-image storage and block storage modes by changing the trigger rate of the pulse-generator. As seen in Fig. 1 ▶, we can recognize that the counting efficiency is gradually decreasing from 100% at 10 Hz to ∼94% at 15 Hz, and there is a sudden drop in the counting efficiency when the trigger rate exceeds 15 Hz. In the trigger rate region that gives rise to the sudden drop in the counting efficiency, it is found that the block storage mode has an efficiency about 1.6 times larger than that of the image-to-image storage mode. In the lower half of Fig. 1 ▶, the measured processing time of the image-to-image storage mode is summarized. In this summary the processing times are shown in milliseconds relative to the reference time of the trigger input. From these measurements the total processing time to complete one cycle of the procedure for the image-to-image storage mode, *i.e.* the time difference between the reference trigger input and re-initialization of the DAQ device, is measured to be 55 ± 10 ms and an acceptable repetition rate of 15 Hz is confirmed to be realised, although the counting-loss is about 6%. At the moment the present system can be operated with a trigger rate of up to 15 Hz and data acquisition without counting-loss can be carried out at a 10 Hz repetition rate, but we are making efforts to further improve the performance of the system.

### 2.3. Photoelectron spectrometer   

We have integrated the present DAQ system with the existing photoelectron spectroscopy apparatus (Takata *et al.*, 2005 ▶, 2007 ▶). The apparatus mainly consists of the hemispherical electron energy analyzer R4000-10kV (VG SCIENTA) mounted on an ultra-high-vacuum system. In addition to the analyzer, a sample manipulator with a motorized *XYZ*Θ stage, a flow-type He cryostat for sample cooling, and two turbomolecular pumps are equipped on the main chamber. This analysis system together with load-lock and preparation chambers are mounted on position-adjustable stages. The vacuum of the main chamber is of the order of 10^−8^ Pa, and the lowest sample temperature that can be achieved is about 20 K.

**Figure 2:**
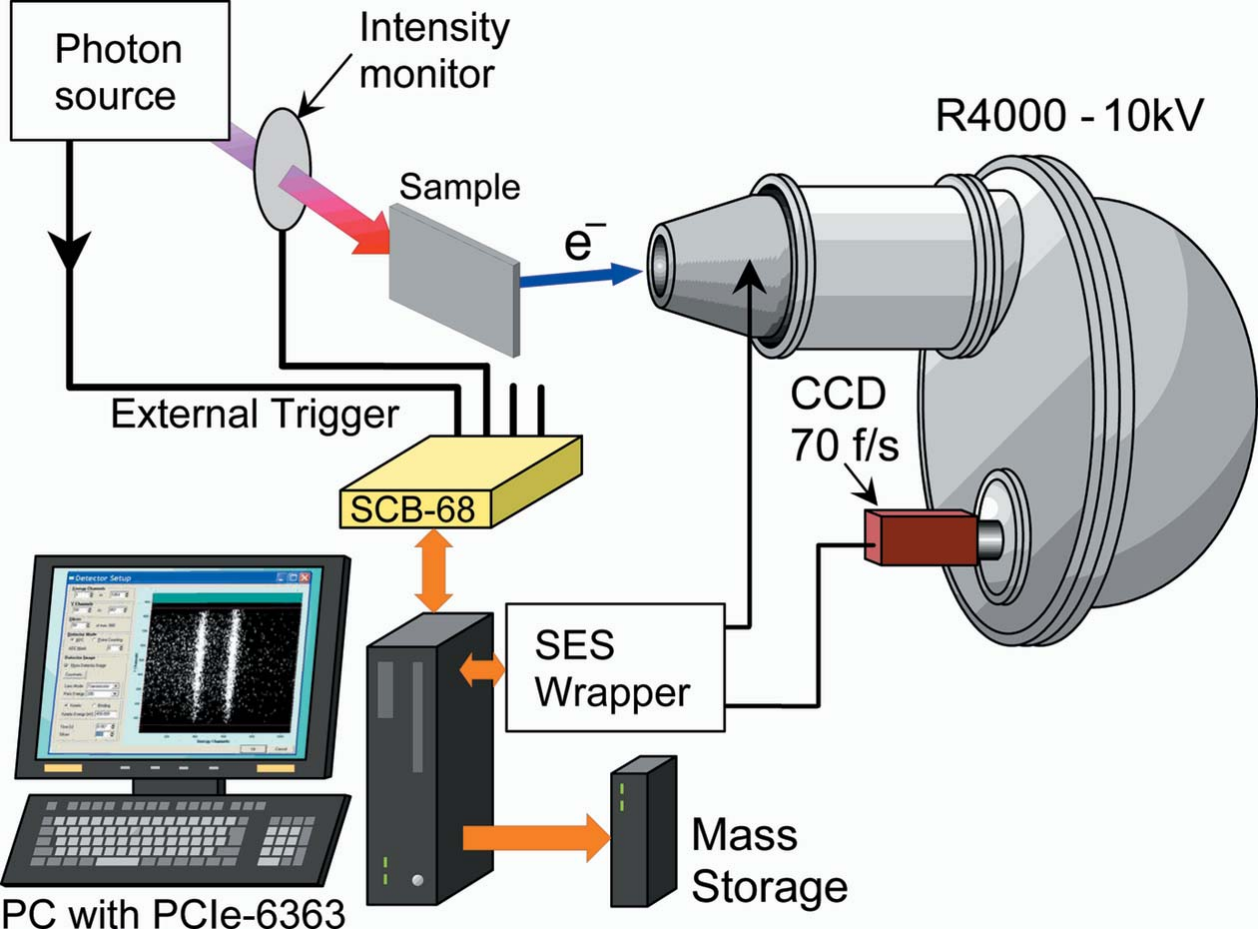
Schematic overview of the single-shot DAQ system for the CCD-based hemispherical electron energy analyzer (VG SCIENTA, R4000-10kV). An external trigger signal is fed into the NI PCIe-6363 DAQ device *via* SCB-68 and starts a procedure to acquire an image of energy-analyzed photoelectrons through the CCD camera. The photoelectron image is immediately transferred to the stacked memory and stored in mass storage, and displayed on the monitor simultaneously. The intensity of the photon beam is also acquired *via* the ADC channel or counter channel of the NI PCIe-6363.

The single-shot DAQ system combined with the photoelectron spectroscopy apparatus, with the aid of an appropriate lens parameter of the analyzer, can be utilized for TR-PES with a variety of wavelengths of the exciting photons, *e.g.* from UV to hard X-rays. Fig. 2 ▶ depicts a typical layout of the measurement system for TR-PES. As shown in Fig. 2 ▶, an external trigger signal from the photon source, *e.g.* the master trigger signal from the accelerator, is fed into the NI PCIe-6363 *via* SCB-68 and issues a command to start a procedure in single-shot image, shot-to-shot image or shot-to-shot sweep mode. An image of energy-analyzed photoelectrons is acquired through the CCD camera and immediately transferred to the stacked memory before being stored in mass storage. At the same time the acquired photoelectron image is displayed on the monitor. Some useful information, such as intensity of the photon beam and information about the wavelength of the exciting photon beam (if possible), can be acquired *via* the ADC channels or counter channel of the NI PCIe-6363.

## 3. Feasibility experiments using synchrotron radiation   

In order to check the feasibility of the present single-shot DAQ system combined with the photoelectron spectroscopy apparatus, we have carried out online test experiments at the soft/hard X-ray undulator beamlines of BL17SU and BL19LXU of SPring-8 (Ohashi *et al.*, 2007 ▶; Senba *et al.*, 2007 ▶; Yabashi *et al.*, 2001 ▶).

In the case of test experiments at soft X-ray beamline BL17SU, the apparatus was installed downstream of the exit slit of the beamline optics. Target materials were chosen to be a thin Au layer vacuum-evaporated onto a copper plate and a La-doped SrTiO_3_ (STO) chip. The beam size on the target was roughly estimated to be 230 µm (H) × 200 µm (V). In between the apparatus and the exit-slit there is a beam diagnostic section, where the incoming intensity of the soft X-ray beam can be monitored *via* the ADC or counter channel of the NI PCIe-6363. The excitation energy of the photon beam was fixed to be 600.2 or 798.5 eV for the Au sample and 598.7 eV for the STO sample, and the bandwidth of the photon beam was about 200 meV. The electron analyzer was set to the condition with a pass energy (*E*_p_) of 200 eV and a slit of 0.5 mm. The emitted photoelectrons or Auger electrons were measured in single-shot image, shot-to-shot image and shot-to-shot sweep modes. The ‘X channel’ of the CCD camera was suitably determined by using a standard sample and a well calibrated incident soft X-ray beam. This is a very important process for calibrating the kinetic energy scale in the shot-to-shot sweep mode. We have also checked the capability of the software for correcting the intensity fluctuations of the exciting photon beam by adjusting the width of the exit slit.

In the case of test experiments at hard X-ray beamline BL19LXU, the apparatus was installed in experimental hutch EH3. The experimental set-up was almost the same as that previously reported (Takata *et al.*, 2007 ▶). A high-resolution, Δ*E* ≃ 60 meV, monochromatic X-ray beam of 7.941 keV was focused onto the STO target. The spot size at the sample position was about 35 µm (H) × 50 µm (V). The set-up of the electron analyzer was the same as for the measurement at BL17SU. The Ti 1*s* HAXPES and *KLL* Auger electron spectra were measured in ordinary sweep mode as reference spectra.

**Figure 3:**
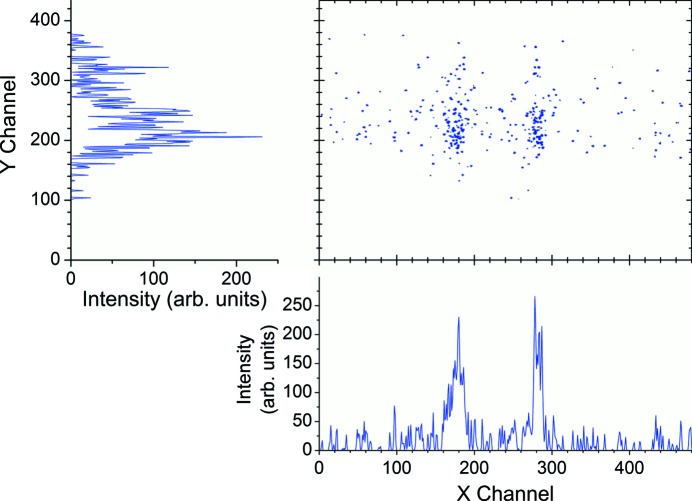
Typical example of a single-shot image (upper-right panel) of Au 4*f* photoelectrons emitted by soft X-ray irradiation onto the thin Au sample. Such an image was sequentially acquired in shot-to-shot sweep mode. The CCD camera was exposed for 14 ms. Both upper-left and lower panels are projected spectra of a two-dimensional single-shot image onto the ‘Y channel’ and ‘X channel (or energy channel)’.

Fig. 3 ▶ shows a typical example of image and projected spectra of Au 4*f*_7/2,5/2_ photoelectrons emitted by 798.5 eV photon irradiation. This image is one of the 201 consecutive images recorded in shot-to-shot sweep mode, in which the kinetic energy is scanned from 705 to 725 eV with 0.1 eV step size. The two-dimensional intensity map (upper-right panel) represents the energy-analyzed photoelectron image accumulated at 14 ms exposure time. The horizontal scale corresponds to the kinetic energy of the photoelectrons, and the vertical scale provides information about the spatial origin of the photoelectrons. Thus the ‘X channel’ projected spectrum (lower panel) gives us the kinetic energy spectrum and the ‘Y channel’ projection gives us the intensity profile as a function of position along the footprint of the synchrotron radiation beam on the sample. Although the image and spectra were recorded as a single-shot image for 14 ms exposure time, the storage ring was operated with the multi-bunch operational condition and thus the image was actually the sum of a large but fixed number of cumulative shots.

**Figure 4:**
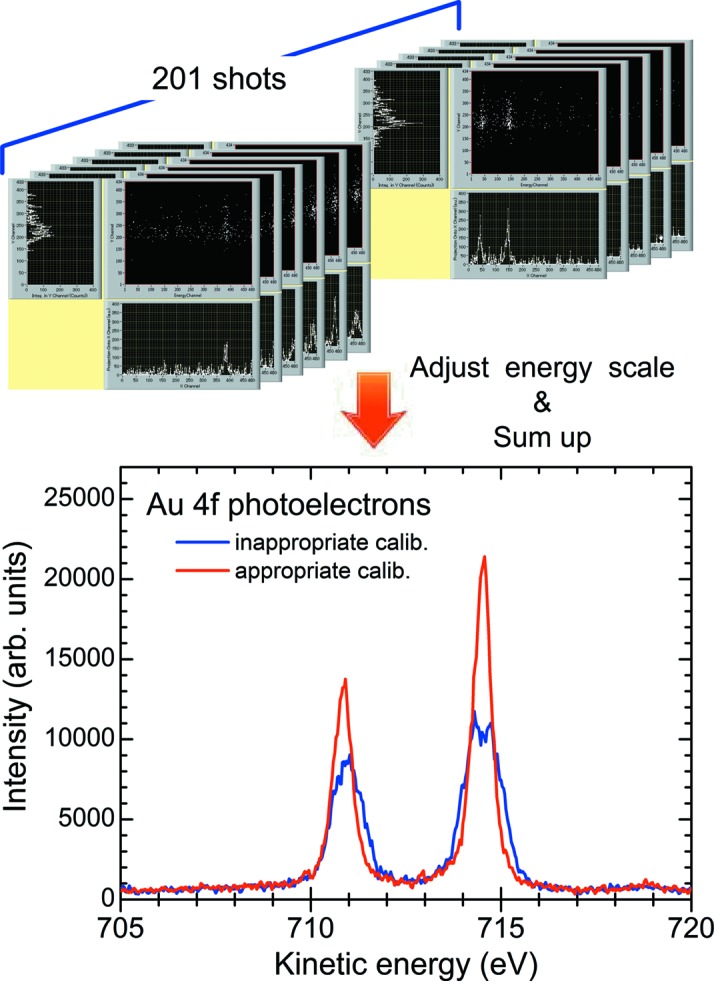
The source image data measured with the shot-to-shot sweep mode (upper-half) and the resultant kinetic energy spectra of Au 4*f* photoelectrons. The spectra were composed of 201 consecutive images. All of the source PE images were recorded as single-shot images (14 ms exposure time) while sweeping the kinetic energy from 705 eV to 725 eV with 0.1 eV step, and their X-projection summed up with adequate calibration. The red solid curve is the resultant Au 4*f* photoelectron spectrum with appropriate calibration. The blue solid curve, on the other hand, is a typical example of a resultant spectrum with inappropriate calibration.

In Fig. 4 ▶ the upper half of the figure shows the source image data measured in the shot-to-shot sweep mode and the lower half represents the resultant kinetic energy spectrum of Au 4*f*_7/2,5/2_ photoelectrons. As mentioned above, 201 spectra were consecutively measured by sweeping the kinetic energy. Each photoelectron image was projected onto the kinetic energy axis, and all the spectra were summed by adjusting the shift, which is inherent to the sweep mode, of energy with suitable calibration. The red solid curve represents the final spectrum summed with appropriate calibration, while the blue solid curve, on the other hand, depicts a typical example with inappropriate calibration.

**Figure 5:**
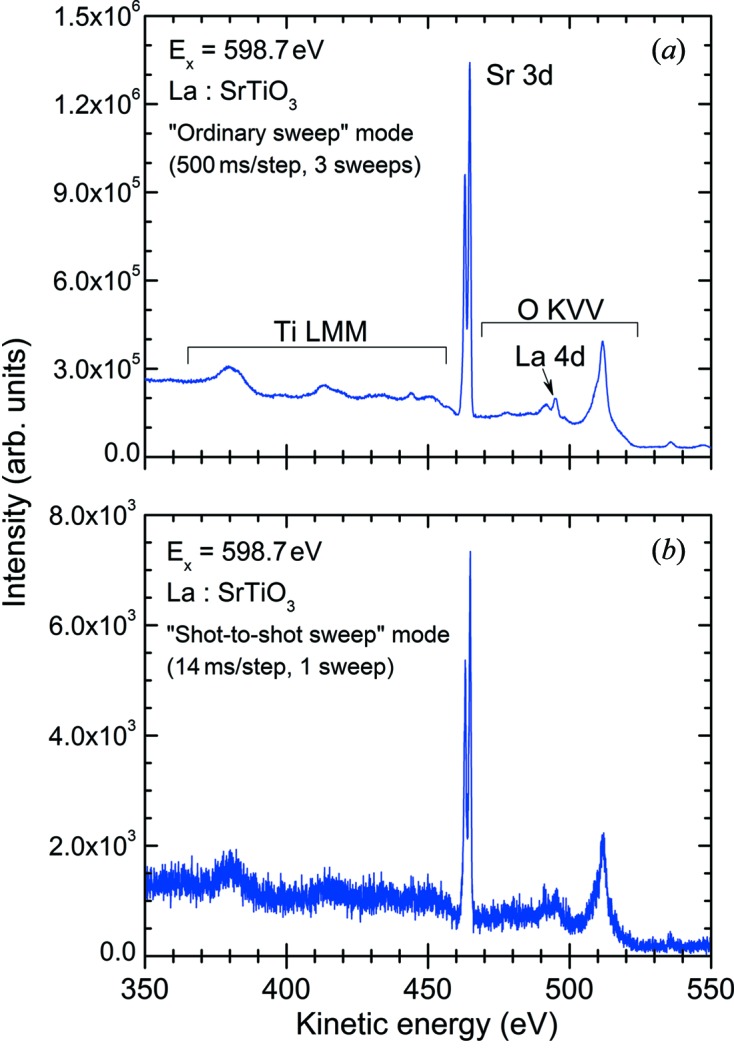
Soft X-ray induced electron spectra from an STO target measured in (*a*) ordinary sweep mode and (*b*) shot-to-shot sweep mode.

We have measured the soft X-ray induced electron spectra from an STO target with different acquisition modes for comparison. Fig. 5 ▶ shows a comparison of the wide-range spectra of STO measured by means of ordinary sweep and shot-to-shot sweep modes. The wide-range spectrum measured in shot-to-shot sweep mode is composed of 2151 consecutive images. In the spectra, pronounced peaks of Sr 3*d* photoelectrons and also the broad structure of Ti *LMM* as well as O *KVV* Auger electrons are observed. The large difference in the statistics comes from the difference in the total exposure time of the CCD. It is shown, however, that the acquired spectra are essentially the same. This result confirms that shot-to-shot sweep mode works properly.

**Figure 6:**
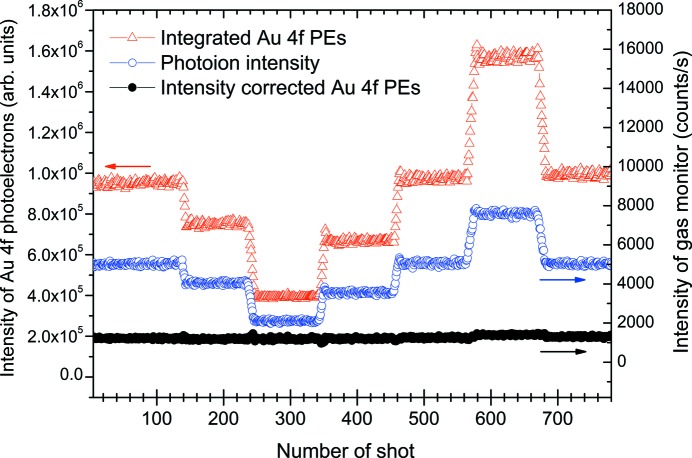
Intensity charts of integrated Au 4*f* photoelectrons (PEs) (red open triangles), photoions (blue open circles) and integrated Au 4*f* PEs intensity corrected by photoion intensity (black closed circles) as a function of shot number. Images for the Au 4*f* PEs were measured for 14 ms exposure time in shot-to-shot image mode using a 600.2 eV soft X-ray synchrotron radiation beam. Intensity jumps in the charts correspond to the change of the width of the exit slit of the synchrotron radiation beamline.

**Figure 7:**
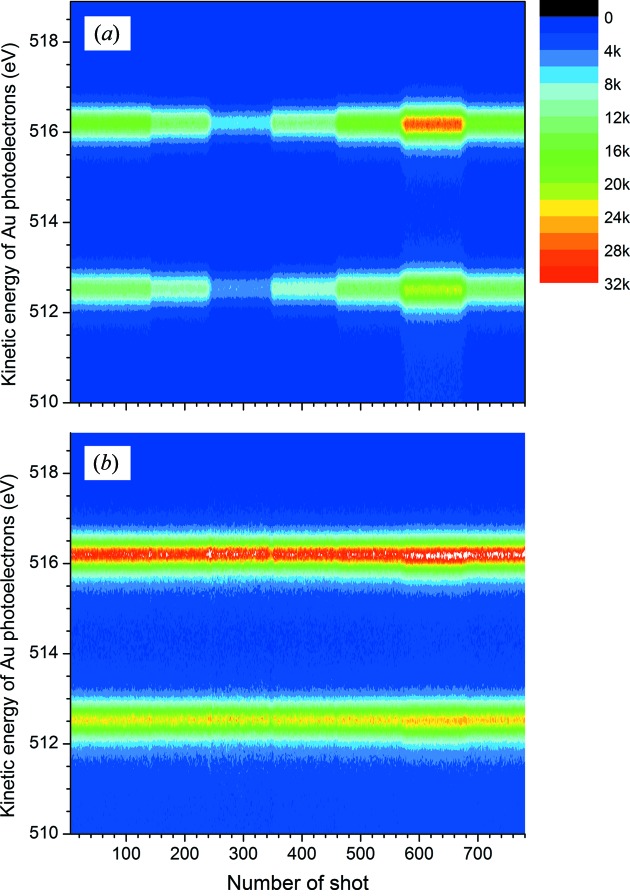
Au 4*f* photoelectrons intensity maps (*a*) before and (*b*) after correction, measured in shot-to-shot image mode as a function of shot number.

Fig. 6 ▶ presents results of a demonstration exhibiting the correction of intensity fluctuations of an exciting photon beam during a measurement for hours in shot-to-shot image mode. The intensity of the photon beam was intentionally changed by adjusting the width of the exit slit and monitored by measuring the photoion intensity at the beam diagnostic section. In Fig. 6 ▶, red open triangles show the integrated Au 4*f* photoelectrons, blue open circles represent the intensity of photoions, and black closed circles show the resultant Au 4*f* photoelectron intensity corrected by using the measured photoion intensity. A small hump seen in the corrected intensity in the region between 580 and 680 shot number is considered to be due to the saturation of the photoion intensity monitor. Figs. 7(*a*) and 7(*b*) ▶ show Au 4*f* intensity maps, before and after correction, as a function of shot number. As can be seen from these figures, the intensity correcting function works correctly. This indicates that shot-to-shot image or shot-to-shot sweep mode can be suitably applied to the measurement of photoelectrons emitted by an intensity fluctuating photon source, such as a SASE-FEL source.

## 4. Application of the system to HAXPES at an X-ray free-electron laser facility   

As mentioned earlier, our motivation to develop the present system is to advance pump–probe-type TR-PES using low-repetition-rate and high-brilliance photon sources. Time-resolved hard X-ray photoelectron spectroscopy (TR-HAXPES) will be one of the targets in possible applications of the present system, since HAXPES has some advantages, *e.g.* surface insensitivity and large probing depth, compared with PES with UV or soft X-ray photon beam (Takata *et al.*, 2005 ▶, 2007 ▶). Thus we have applied the present single-shot DAQ system to HAXPES at the SACLA XFEL facility (Ishikawa *et al.*, 2012 ▶).

The apparatus was installed in experimental hutch EH2 of BL3. The electron analyzer was set to the condition with *E*_p_ = 200 eV and a slit of 4 mm so as to increase the efficiency. The target material was La-doped STO and we measured the Ti 1*s* HAXPES spectra in shot-to-shot image and ordinary sweep modes using 8 keV monochromatic XFEL beam with Δ*E* ≃ 1 eV. Under these conditions the FWHM of the Ti 1*s* HAXPES peak can be theoretically estimated to be about 2.7 eV if we employ a natural width value of 0.8 eV (Krause, 1979 ▶) for the Ti 1*s* hole state. The typical intensity of the XFEL beam obtained from the double-crystal monochromator was about 3.85 µJ per pulse, *i.e.* 3 × 10^9^ photons per pulse, with the spot size at the sample position of about 0.6 mm in diameter.

**Figure 8:**
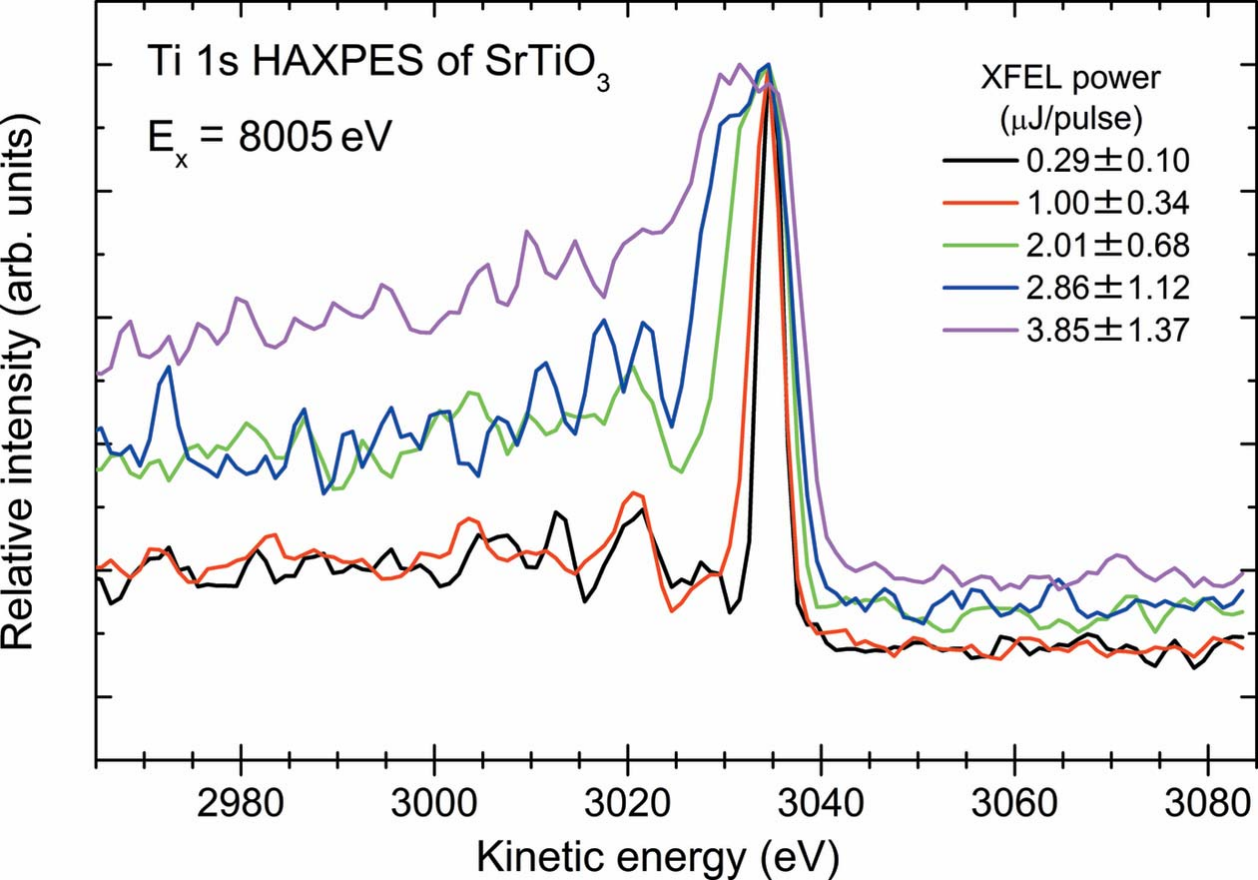
Ti 1*s* HAXPES spectra of STO measured using an 8 keV monochromatic XFEL beam at SACLA as a function of XFEL power.

As shown in previous studies (Pietzsch *et al.*, 2008 ▶; Hellmann *et al.*, 2010 ▶, 2012 ▶), the photoelectron spectrum will be broadened and energy-shifted due to the FEL-induced space-charge effects. Although the statistics are rather poor because of the small photoionization cross section at 8 keV, we have confirmed that the Ti 1*s* photoelectron spectra show significant tailing toward the lower kinetic energy side as the XFEL power increases, as shown in Fig. 8 ▶. This tailing is supposed to be due to the space-charge effects caused by preceding electron packets of Sr 2*p* photoelectrons as well as Ti *KLL* Auger electrons, which have higher kinetic energies than the Ti 1*s* photoelectron. The Ti 1*s* photoelectron packet is therefore pushed back due to the repulsive force created by these preceding electron packets. The broadening, on the other hand, is considered to be due to the sum of intra-packet and inter-packet Coulomb repulsive forces.

**Figure 9:**
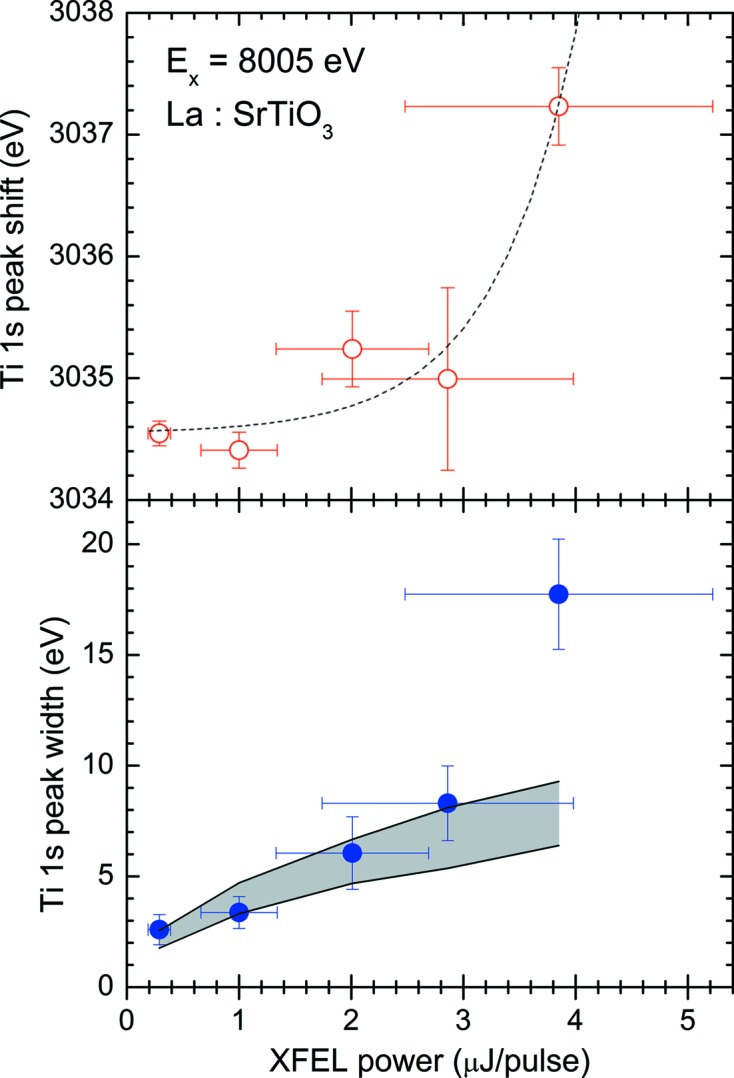
Peak shift (top) and peak width (bottom) of Ti 1*s* photoelectrons, observed as a function of XFEL power. Both the peak shift and the peak width are determined by means of a least-squares fitting procedure. The dashed line in the upper figure is an exponential fit to the measured shifts. The shaded area represents the space-charge-induced kinetic energy bandwidth calculated by the mean-field model.

In order to determine the appropriate XFEL power for the HAXPES measurements with acceptable space-charge effects, we have carried out a simple analysis of all the spectra shown in Fig. 8 ▶ after subtracting Shirley’s type background (Shirley, 1972 ▶) from each spectrum. The peak position of the Ti 1*s* core-level for each condition of XFEL power was determined by the least-squares fitting procedure by employing the asymmetric double sigmoidal function. The results are plotted together with an exponential fit (dashed curve) in the upper half of Fig. 9 ▶. On increasing the XFEL power, the peak centers shift to the higher kinetic energy side and the widths of the lower kinetic energy side become larger giving rise to significant tailing. As mentioned above, this tailing tendency may be attributed to space-charge effects in which the preceding electron packets repel back the Ti 1*s* photoelectron on the way to the analyzer. The peak width of the Ti 1*s* core-level, on the other hand, for each XFEL power was also determined by the fitting procedure as shown in the lower half of Fig. 9 ▶. As described above, the bare peak width of the Ti 1*s* core-level for the present experimental conditions can be theoretically calculated to be about 2.7 eV. Thus the measured width of 2.6 eV for the lowest XFEL power is consistent with the theoretical peak width. We have then applied the ‘mean-field model’ (Siwick *et al.*, 2002 ▶) of femtosecond electron packet propagation to estimate the energy broadening of the Ti 1*s* peak width. According to the criterion discussed in the previous work (Hellmann *et al.*, 2009 ▶), the Ti 1*s* photoelectron packet can be considered as a quasi-two-dimensional disk in front of the sample surface. The mean-field model predicts a square-root dependence of the space-charge broadening on the number of electrons per pulse. Thus we estimate the number of emitted photoelectrons per pulse by using the following simple expressions,
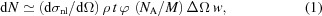


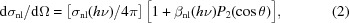
where ρ is the density of STO (g cm^−3^), *t* is the thickness of the target (cm), ϕ is the number of photons per pulse, *N*_A_ is Avogadro’s number (mol^−1^), *M* is the molecular weight (g mol^−1^), ΔΩ is the solid angle subtended by the electron analyzer (sr), *w* is the atomic weight % of Ti in STO, σ_nl_ is the Ti 1*s* photoionization cross section (cm^2^) (Scofield, 1973 ▶), β_nl_ is the energy-dependent asymmetry parameter, *P*_2_(cos θ) = (1/2)(3 cos^2^θ − 1), and θ is the angle between the polarization vector and photoelectron direction. Substituting the values for our experimental conditions as well as relevant information for the target materials in the above expression, we have estimated the space-charge broadening. For example, when the XFEL power is 1.0 µJ per pulse the resultant number of emitted photoelectrons per pulse can be calculated to be about 165, which gives rise to a space-charge-induced kinetic energy bandwidth of about 4 eV. Similarly, the peak width has been calculated with the corresponding XFEL power for each case shown in Fig. 8 ▶, and the results are shown as the shaded area in the lower half of Fig. 9 ▶. As can be seen in Fig. 9 ▶, the peak widths for lower XFEL power are reasonably explained by the mean-field model. Only for the highest XFEL power, on the other hand, does the measured peak width show a deviation from the model. This deviation might be due to the emergence of the influence of the inter-packet Coulomb repulsive force. In order to fully understand such femtosecond electron packet propagation dynamics, we probably need to consider a more rigorous simulation taking into account inter-packet Coulomb repulsion.

**Figure 10:**
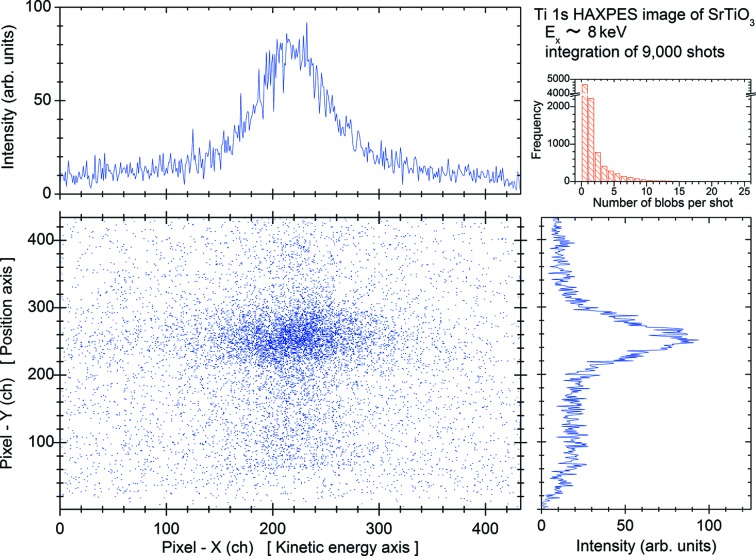
Ti 1*s* photoelectron spectral image (lower-left) and its projected spectra (upper-left and lower-right) measured in shot-to-shot image mode. The inset (upper-right) represents the statistics of the number of recorded detector hits for each shot.

**Figure 11:**
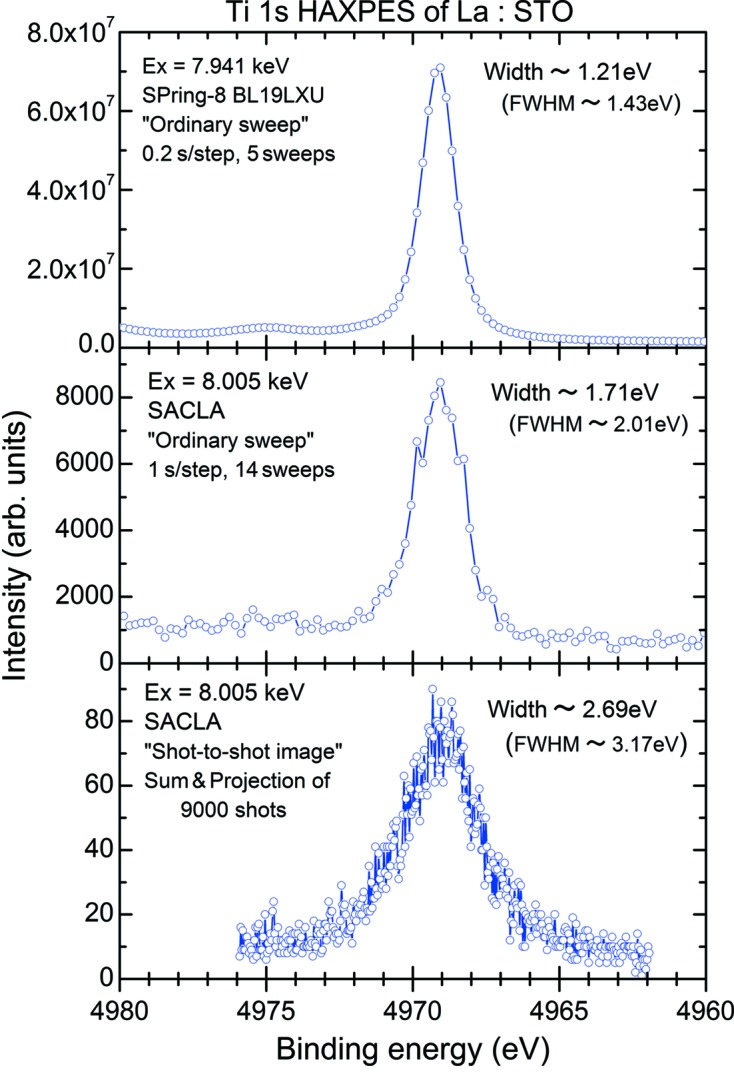
Comparison of Ti 1*s* HAXPES spectra measured under different conditions. Widths indicated in the figure were determined by means of the least-squares fitting procedure employing the Gaussian function. (Top) Ordinary sweep mode, BL19LXU of SPring-8 with *E*_p_ = 200 eV, slit = 0.5 mm and photon bandwidth ≃ 60 meV. (Middle) Ordinary sweep mode, BL3 of SACLA with *E*_p_ = 200 eV, slit = 4 mm and photon bandwidth ≃ 1 eV. (Bottom) Shot-to-shot image mode, BL3 of SACLA.

In order to avoid these space-charge effects, the intensity of the XFEL beam had to be reduced to 7.6% (∼0.29 µJ per pulse) of the monochromator output by inserting the Al filter. Under this condition we have tried to acquire the core-level photoelectron image in shot-to-shot image mode. Fig. 10 ▶ shows the integrated Ti 1*s* photoelectron image of 9000 shots and its projections onto the ‘X channel (or energy channel)’ and ‘Y channel (or position channel)’. In the upper-right of the figure the histogram showing the number of detector hits per shot is also indicated. According to this histogram we found that about half of the total shots did not contain any measured photoelectrons. The number of photoelectrons for each shot is quite a lot less, *i.e.* the averaged detector hits for 9000 shots is only about 1.2, so that we have measured all the kinetic energy spectra induced by the XFEL beam by means of ordinary sweep mode with long exposure time so as to smooth out intensity fluctuations. Under the same conditions, the Ti 1*s* HAXPES spectrum has been measured in ordinary sweep mode at SACLA and is compared with that measured in the same mode at BL19LXU of SPring-8. In Fig. 11 ▶ the spectra in the vicinity of the Ti 1*s* core-level are represented for comparison. The top panel indicates the spectrum measured at BL19LXU and the middle panel depicts the resultant spectrum measured at SACLA. Although the statistics of the HAXPES spectrum of SACLA are smaller, it definitely shows a nice spectrum which was measured in ∼1 h. In the bottom panel the Ti 1*s* HAXPES spectrum measured in shot-to-shot image mode is also shown for comparison. As can be seen from Fig. 11 ▶, the Ti 1*s* HAXPES spectra measured in ordinary sweep mode, *i.e.* both of the spectra shown in the top and middle panels, reflect the total instrumental energy resolution including the X-ray bandwidth. In the case of the Ti 1*s* HAXPES spectrum measured in shot-to-shot image mode, the analyzer was operated in fixed mode so that the resultant spectral width becomes larger compared with the case of the energy-sweep mode. These results confirm the capability for core-level HAXPES spectra of condensed matter induced by the XFEL beam.

**Figure 12:**
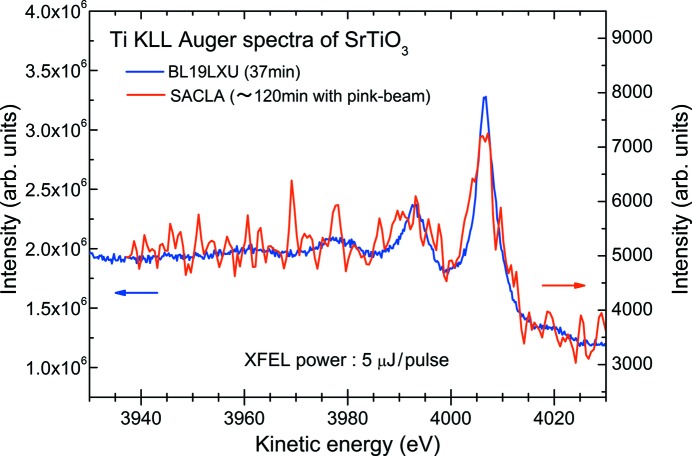
Comparison of Ti *KLL* Auger electron spectra measured at SPring-8 BL19LXU (blue curve) and SACLA (red curve). The non-monochromatic XFEL beam of 250 µJ per pulse was reduced to 2% intensity, *i.e.* 5 µJ per pulse, by inserting the Al filter.

Finally, with the aid of the fact that the Auger electron spectrum can be acquired using white beam, we have tried to measure the Ti *KLL* Auger electron spectrum of STO by using the non-monochromatic XFEL beam, the so-called pink-beam, centered at 8 keV with an intensity of 250 µJ per pulse. Fig. 12 ▶ represents the Ti *KLL* Auger electron spectra measured at SACLA (red curve), with that obtained at BL19LXU (blue curve) for comparison. The accumulation time was 500 ms per step and the Auger electron spectrum at SACLA is just the result of one sweep. The power of the XFEL pink-beam was reduced to be 2%, *i.e.* 5 µJ per pulse. We have found that the Auger electron spectrum is significantly distorted due to the space-charge effects so that we need to reduce the intensity of the XFEL beam by about two orders of magnitude. We come to the fact that spectroscopic studies of condensed matter with core-level Auger electron spectroscopy using XFELs is achievable. However, if we try to perform the time-resolved measurement, especially for the temporal evolution measurement of the transient electronic state by means of pump–probe-type Auger electron spectroscopy, it is rather time-consuming.

## 5. Perspective   

For the TR-HAXPES using the shot-to-shot measurement scheme, the repetition rate of SACLA is suitable for the present DAQ system. At the moment SACLA is operated with a 20 Hz repetition rate, so that the shot-to-shot image or shot-to-shot sweep mode of the system is applicable by combining with the pulse selector (Kudo *et al.*, 2009 ▶). The minimum exposure time of the present CCD camera is 14 ms, long enough to acquire all the electrons in the single shot. The pulse duration of the XFEL pulse from SACLA is measured to range between 4.5 and 31 fs (Inubushi *et al.*, 2012 ▶). The ultimate resolution of the time-resolved pump–probe measurement will be around several tens of femtoseconds, if we can tune the optical pump laser to have a pulse duration comparable with the probe pulse. By tuning the delay between the optical pump and XFEL probe pulses, the temporal evolution of the intrinsic electronic structure of various materials induced by the pump laser will be probed by the XFEL pulse. We have recently carried out a TR-HAXPES experiment at SACLA using the pump–probe technique. The theoretical analyses of the experimental results are in progress and will be published elsewhere.

Here we have opened up the opportunity of studying the transient electronic structure of condensed matter on a sub-picosecond time scale by TR-HAXPES at the SACLA XFEL facility. Although the measurement of the valence band seems to be difficult because of the small photoionization cross section, the ultrafast core-level dynamics can be extensively studied using the present single-shot DAQ system combined with the high-resolution hemispherical electron energy analyzer. In the near future, when the SACLA is available for providing a soft X-ray FEL beam, the measurement of the transient electronic structure in the valence band will be challenged.

Furthermore, we are also planning to carry out TR-HAXPES at BL19LXU of SPring-8. Although the ultimate time resolution is restricted to 40 ps, which is the pulse duration of the synchrotron radiation beam from SPring-8, a higher repetition rate of 1 kHz with negligible space-charge effects would be applicable to the TR-HAXPES experiment to study the fast dynamics on a sub-nanosecond time scale.

## 6. Summary   

In conclusion, we have developed a single-shot CCD-based data acquisition system combined with a high-resolution hemispherical electron energy analyzer. It can be triggered by an external signal to carry out the shot-to-shot measurement for hard X-ray photoelectron spectroscopy (HAXPES) using a low-repetition-rate and high-brilliance photon source such as a free-electron laser source. The system has been successfully characterized with offline and online tests of feasibility experiments using soft/hard X-ray photoelectron spectroscopy at high-brilliance photon source. The results of these characterizations satisfactorily demonstrate its performance of the shot-to-shot measurement scheme for near-future time-resolved HAXPES using XFEL sources.
